# Papilledema as the Sole Manifestation of Neuroborreliosis

**DOI:** 10.1155/2021/5565900

**Published:** 2021-05-30

**Authors:** Kaitlyn May, Shankar Upadhyayula

**Affiliations:** ^1^Department of Pediatrics, Akron Childrens Hospital, Akron, OH, USA; ^2^Division of Infectious Diseases, Akron Childrens Hospital, Akron, OH, USA

## Abstract

Lyme disease is reportable, and approximately 30,000 cases/year are notified to the local and state health departments. However, other estimates based on insurance records suggest there are close to 476,000 cases/year that are diagnosed and treated. In addition to a large burden of illness, areas where Lyme disease is common are expanding. Therefore, clinicians should be aware of uncommon presentations of this condition. We describe the case of a 5-year-old girl who presented with papilledema as an isolated manifestation of Lyme disease. Of note, her ocular symptoms were intermittent and worse when tired. In endemic areas, Lyme disease must be considered in the differential diagnosis for patients presenting with isolated ophthalmic findings even outside the usual Lyme season.

## 1. Introduction

Lyme disease is the most common vector-borne illness in the United States (US) and is endemic in Northeast Ohio [1, 2]. The number of diagnosed cases appears to be increasing [3]. Our own institutional surveillance identified a 5-fold increase in Lyme disease cases from 2014 to 2020 (unpublished data). The causative organism in the US is *Borrelia burgdorferi* sensu stricto (hereafter referred to as *B. burgdorferi*), which is spread by the infected blacklegged tick [4]. In contrast, European Lyme is caused primarily by *B. garinii* and *B. afzelii* species. Lyme disease can affect multiple organ systems including the skin, joints, heart, eyes, and nervous system with neurologic complications reported in 10% of cases [4–6]. The most common neurologic manifestation in children is headache followed by facial nerve palsy [5]. Subacute lymphocytic meningitis is also frequently described [7]. Meningitis, intracranial hypertension, and papilledema occur more commonly in children than adults [8]. Neuroborreliosis seems more common in Europe likely related to different species of Lyme bacteria. The most common manifestation in Europe is painful meningoradiculitis (Bannwarth's syndrome), while acute myelitis is rare (<5% of patients). Painful neuroradicular symptoms and other cranial nerve involvement besides the facial nerve are rare in the US [9]. When ocular involvement is reported in association with Lyme disease [10], there are usually additional symptoms present [8]. We report the case of a 5-year-old female with papilledema as an isolated finding of neuroborreliosis.

## 2. Case Report

**Figure 1:**
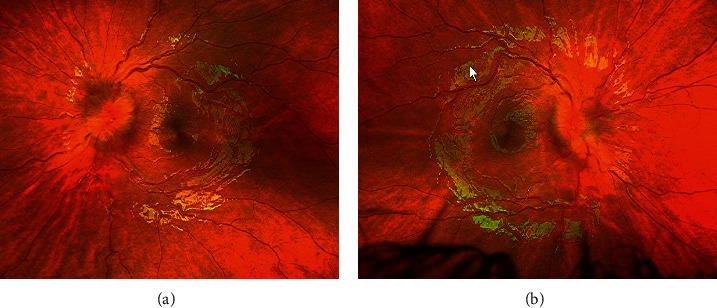
Disc edema at presentation. (a) Left eye fundus exam. (b) Right eye fundus exam.

A previously healthy 5-year-old Caucasian female presented with photophobia, diplopia, eye pain, and internal deviation of the right eye. Symptoms started 10 days prior to her initial evaluation and were reported to be intermittent and worse when tired. She had no associated headaches or facial palsy. She was initially evaluated by an optometrist who referred her to ophthalmology due to the concern for possible optic nerve swelling. On fundoscopic exam, she was found to have bilateral optic disc swelling with elevation, blurring, and large vessels crossing elevated margins consistent with papilledema (Figure 1). Visual acuity was 20/20, and bilaterally pupillary reactions were normal. Due to the concern for elevated intracranial pressure, she was directed to the emergency department (ED).

Reportedly, the patient had a rash on the right side of her face a month prior that was described as nonitchy and nonpainful. In the ED, the patient had normal vitals and nonfocal neurologic exam. Computerized tomographic (CT) scan of the head without contrast was normal. Magnetic resonance imaging (MRI) showed bilateral papilledema, normal venous phase study, and normal appearance of the brain tissue. Cerebrospinal fluid (CSF) opening pressure was 17 cm of H_2_O (normal range: 10–28). CSF had 29 white blood cells/high-power field (normal range: 0–7) with lymphocytic predominance (77%). Protein and glucose were within normal limits. CSF cultures were sterile. Multiplex PCR panel (Biofire FilmArray®) on the cerebrospinal fluid did not identify any of the amplified targets. Blood tests including metabolic panel, complete blood count, thyroid-stimulating hormone, lactate dehydrogenase, uric acid, ferritin, and C-reactive protein were within normal limits. Erythrocyte sedimentation rate was 26 mm/hr (normal: 0–20). Antinuclear antibody, antineutrophilic cytoplasmic antibody, and antimyelin oligodendrocyte glycoprotein were all negative. Arboviral antibody panel from serum (including West Nile, Eastern, and Western equine encephalitis and LaCrosse all of which are reported in Ohio) was negative. *Bartonella henselae* and *Bartonella quintana* serologies were negative. Lyme serology was positive, and immunoblot confirmed positive IgG bands 6/10 (p66, p45, p41, p39, p23, and p18) and IgM bands 3/3 (p41, p39, and p23). CSF Lyme PCR and CSF/serum antibody index were not done. She was started on doxycycline and completed a total of 21 days of therapy. Double vision and eye pain resolved quickly after the initiation of antibiotics. She did not require any additional therapies including steroids. Follow-up appointment with ophthalmology one month after diagnosis revealed improving disc edema.

## 3. Discussion

Isolated papilledema is a rare manifestation of Lyme disease that typically presents as diplopia [6]. Other ocular manifestations reported in Lyme disease include conjunctivitis, choroiditis, or papillitis [10, 11]. Papilledema is typically a delayed diagnosis, and ocular manifestations are reported to occur over a wide time range—1.5 weeks to 51 months following the initial infection [5]. Therefore, Lyme disease, as a cause for diplopia, is often missed. Lyme disease is diagnosed by serologic testing to identify antibodies to *B. burgdorferi* [9]. While CSF studies are not required for diagnosis, they may provide supportive evidence. The most common CSF abnormality is mildly elevated protein along with lymphocytic pleocytosis [2, 5]. The yield of PCR testing on cerebrospinal fluid samples from patients with neuroborreliosis is too low to be useful in excluding this diagnosis [12]. The presence of *B. burgdorferi*-specific antibodies in the CSF with evidence of intrathecal production is the traditional diagnostic gold standard, but has limitations [9], and in our experience, it is not always pursued in the US. The differential diagnosis for children presenting with diplopia is very broad and includes intracranial mass, idiopathic intracranial hypertension, infectious etiologies, head trauma, or impaired ventricular flow.

Our patient was a previously healthy 5-year-old female who presented with diplopia, photophobia, internal deviation of the right eye, and eye pain. She was found to have bilateral optic disc swelling. Extensive workup showed positive Lyme serology with immunoblot confirmation. She did not recall any tick exposure. She did have a nonpruritic, nontender rash on the right side of her face one month prior, but as is often the case, this may have been confused for other nonspecific rashes.

PubMed review (1998–present) of ocular manifestations in Lyme disease in children revealed the following (please see Table 1 for a summary).

Rothermel et al. [11] reviewed four cases of children with optic neuropathy associated with Lyme disease. An 8-year-old male presented with headache, fatigue, and vision abnormalities. Visual symptoms were reported 8 weeks after the onset of headaches. He was found to have bilateral papillitis and positive Lyme serologies. A 16-year-old male was treated for Lyme disease with antibiotics after presenting with arthritis of the left knee, and six months later, he developed blurred vision of the left eye and was found to have unilateral optic neuritis with positive Lyme serology. A 13-year-old female presented with headache, nausea, and low-grade fever, and three weeks later, she developed horizontal diplopia and neck pain. She had bilateral optic disc swelling and positive Lyme serology. An 11-year-old male presented with severe headaches, nausea, vomiting, and fever, and 2.5 weeks later, he developed diplopia which progressed to vision loss by 7.5 weeks from symptom onset. He was found to have bilateral optic disc edema and optic atrophy in the setting of positive Lyme serologies. All of the children had improvement of symptoms with appropriate therapy although the 11-year-old did have persistent visual impairment.

Ezequiel et al. [7] reported a case of a 9-year-old male who presented with headache, phonophobia, and photophobia but no visual changes. He was found to have bilateral papilledema on exam and an elevated opening pressure during lumbar puncture. His serum *B. burgdorferi* antibodies were positive on ELISA. Papilledema resolved with antibiotic therapy.

Kan et al. [4] reported a case of an 8-year-old female who presented with headache, vomiting, and diplopia. Exam revealed bilateral papilledema and sixth cranial palsy. She was also found to have enhancement of the dura on MRI consistent with the inflammatory reaction. She had elevated opening pressure during lumbar puncture. Both her CSF and serum were positive for *B. burgdorferi* antibodies on ELISA and immunoblot. After antibiotic treatment, her symptoms improved, although she had residual sixth cranial nerve palsy.

Similar to previous reports, our patient also had the symptoms of diplopia, photophobia, and eye deviation, but the unusual feature of this case is isolated ocular symptoms. All of the patients described above had additional symptoms including headache or low-grade fever that were helpful in guiding workup and diagnosis. Given our patient's young age, there is the possibility that she had difficulty reporting headaches. However, one would expect that since she was able to articulate her visual symptoms, she would be able report headaches as well.

**Figure 2:**
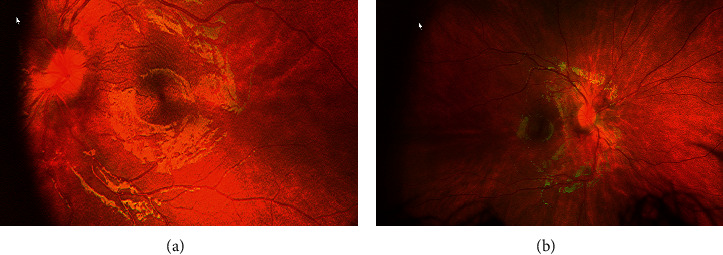
Improved disc edema on follow-up. (a) Left eye fundus exam. (b) Right eye fundus exam.

Our patient was treated with a 21-day course of doxycycline therapy. This choice was based on the recommendation that “A growing body of evidence suggests that oral doxycycline is effective for the treatment of Lyme meningitis and may be used as an alternative to hospitalization and parenteral ceftriaxone therapy in children who are well enough to be treated as outpatients.” [12, 13] Her symptoms resolved with antibiotic therapy. After the completion of therapy, a repeat fundoscopic exam demonstrated improvement in optic disc edema (Figure 2).

## 4. Conclusion

Lyme disease must be considered in the differential diagnosis of papilledema for patients living in endemic areas (not only during the Lyme season). The significance is even greater when one recognizes that all the reported patients improved with antimicrobial treatment.

## Figures and Tables

**Table 1:** Summary of the literature review of ocular manifestations of Lyme disease.

Citation	Patient details	Initial symptoms	Time from initial symptoms to ocular symptoms (weeks)	Ocular symptoms	Ocular signs	Antibody to *B. burgdorferi*	Antibody to *B. burgdorferi*	Opening pressure (cm H_2_O)	CSF white cells	Brain MRI findings	Treatment	Outcome
						Bands on WB	CSF/serum ratio					
Rothermel et al. [11]	An 8-year-old male	Headache	8	Decreased vision	Swollen discs with elevation of the retina	10/10 IgG bands	Not done	Normal	0	Normal	Ceftriaxone	Resolution of symptoms
	A 16-year-old male	Knee arthritis	28	Blurred vision of the left eye	Swollen optic disc, central scotoma	8/10 IgG bands	Not done	Normal	<10	Normal	Ceftriaxone	Resolution of symptoms
	A 13-year-old girl	Fever, headache, and neck pain	3	Horizontal diplopia and eye pain	Bilateral papilledema, sixth nerve palsy, and decreased visual acuity	7/10 IgG bands	IgG 1.42	60	82	Normal	Ceftriaxone	Resolution of symptoms
	An 11-year-old male	Rash, headaches, vomiting, and fever	2.5	Double vision	Photophobia and bilateral sixth and seventh nerve palsies	8/10 IgG bands	<1 for IgG, IgM, and IgA	57	3	—	Ceftriaxone and methylprednisolone	Improvement in symptoms with residual sixth nerve palsy

Ezequiel et al. [7]	A 9-year-old male	Headache, pallor, photophobia, and phonophobia	—	—	Papilledema	Positive	Positive IgG and IgM	50	30	Normal	Ceftriaxone	Resolution of symptoms

Kan et al. [4]	An 8-year-old female	Headaches, vomiting, and diplopia	—	Diplopia	Papilledema and left sixth nerve palsy	Positive	Positive CSF antibodies	32	115	Dural enhancement	Ceftriaxone and acetazolamide	Papilledema resolved, and mild sixth nerve palsy remained

## Data Availability

No additional data were available to support this manuscript.

## References

[1] https://www.cdc.gov/lyme/index.html

[2] Eppes S. C., Nelson D. K., Lewis L. L., Klein J. D. (1999). Characterization of lyme meningitis and comparison with viral meningitis in children. *Pediatrics*.

[3] Dumic I., Severnini E. (2018). “Ticking bomb”: the impact of climate change on the incidence of lyme disease. *Canadian Journal of Infectious Diseases and Medical Microbiology*.

[4] Kan L., Sood S. K., Maytal J. (1998). Pseudotumor cerebri in lyme disease: a case report and literature review. *Pediatric Neurology*.

[5] Belman A. L., Iyer M., Coyle P. K., Dattwyler R. (1993). Neurologic manifestations in children with North American lyme disease. *Neurology*.

[6] Kauffmann D. J., Wormser G. P. (1990). Ocular lyme disease: case report and review of the literature. *British Journal of Ophthalmology*.

[7] Ezequiel M., Teixeira A. T., Brito M. J., Luis C. (2018). Pseudotumor cerebri as the presentation of lyme disease in a non-endemic area. *BMJ Case Reports*.

[8] Sibony P., Halperin J., Coyle P. K., Patel K. (2005). Reactive Lyme serology in optic neuritis. *Journal of Neuro-Ophthalmology*.

[9] Mygland Å., Ljøstad U., Fingerle V., Rupprecht T., Schmutzhard E., Steiner I. (2010). EFNS guidelines on the diagnosis and management of European lyme neuroborreliosis. *European Journal of Neurology*.

[10] Winterkorn J. M. S. (1990). Lyme disease: neurologic and ophthalmic manifestations. *Survey of Ophthalmology*.

[11] Rothermel H., Hedges T. R., Steere A. C. (2001). Optic neuropathy in children with lyme disease. *Pediatrics*.

[12] Kimberlin D. W., Brady M. T., Jackson M. A. (2018). *Red Book (2018): Report of the Committee on Infectious Diseases*.

[13] Lantos P. M., Rumbaugh J., Bockenstedt L. K. (2021). Clinical practice guidelines by the infectious diseases society of America (IDSA), American academy of neurology (AAN), and American college of rheumatology (ACR): 2020 guidelines for the prevention, diagnosis, and treatment of lyme disease. *Arthritis Care & Research*.

